# Imaging metabolic heterogeneity in cancer

**DOI:** 10.1186/s12943-015-0481-3

**Published:** 2016-01-06

**Authors:** Debanti Sengupta, Guillem Pratx

**Affiliations:** Stanford University School of Medicine, A226 Building A, 1050 Arastradero Road, Palo Alto, CA 94304 USA

**Keywords:** Metabolic heterogeneity, Imaging, Cancer metabolism

## Abstract

As our knowledge of cancer metabolism has increased, it has become apparent that cancer metabolic processes are extremely heterogeneous. The reasons behind this heterogeneity include genetic diversity, the existence of multiple and redundant metabolic pathways, altered microenvironmental conditions, and so on. As a result, methods in the clinic and beyond have been developed in order to image and study tumor metabolism in the in vivo and in vitro regimes. Both regimes provide unique advantages and challenges, and may be used to provide a picture of tumor metabolic heterogeneity that is spatially and temporally comprehensive. Taken together, these methods may hold the key to appropriate cancer diagnoses and treatments in the future.

## Background

Observations made by Otto Warburg in 1924 have changed both our understanding of cancer as well as the methods that we use to diagnose this disease. Warburg discovered that cancer cells display a particular metabolic signature that he hypothesized was due to greater glucose consumption by cancer cells than healthy cells. At the time, he believed that the reason for cancer was this elevation of metabolism, leading to what is now known as the Warburg hypothesis, which states that the cause of cancer is inefficient cellular respiration. Warburg’s observations are now thought to be a downstream effect rather than the cause of cancer [1], although the Warburg hypothesis itself has come under renewed scrutiny with the discovery that a number of upregulated oncogenes do in fact impact key metabolic pathways [2]. Since the discovery of the Warburg effect, cancer metabolism has been intensively studied, and has even become the basis of key diagnostic imaging platforms for cancer detection and diagnosis. However, cancer metabolism is neither as homogenous nor as reproducible as initially suspected. Rather, the metabolic activity of cancer cells is a complex, heterogeneous, and nuanced process that may be key to successful treatment.

### Metabolic heterogeneity in cancer

Warburg observed that cancer cells that are rapidly proliferating tend to utilize aerobic glycolysis as opposed to oxidative phosphorylation. It is commonly considered that glycolysis is the less efficient, but often faster, metabolic method, and the uncontrolled growth of cancer is often characterized by the presence of glycolytic cells. The cause for this upregulation of glycolysis has not thus far been conclusively elucidated [3]; hypotheses include the presence of hypoxic conditions in pre-malignant lesions [4], microenvironmental adaptations [5], and the conferral of a biosynthetic advantage [6]. Glycolytic cells are considered ‘hypermetabolic’ because they tend to take up much more glucose than surrounding, healthy tissue, and also tend to proliferate rapidly. While many types of cancer cells demonstrate overexpressed glycolytic genes [7], not all rapidly proliferative cancer cells are glycolytic. This observation is reversed in several cancer types, including ablation-resistant pancreatic cancer cells [8], certain breast cancer models [9], and in B-cell lymphoma [10].

A plethora of purported oncogenes are mutated across different cancer types, which may impact metabolism. These include Akt [11], HIF1 [12], c-myc [13], oct1 [14], p53 [15], and others [16]. It is important to note, however, that all cancerous cells that make up a tumor do not behave in a uniform fashion since genes can be regulated at the single cell level, and not all cancer cells demonstrate increased proliferation relative to healthy cells. Multiple, and occasionally redundant, metabolic pathways can generate an atypical metabolic phenotype [17]. While specific pathways have been implicated in cancer metabolism, it has also been demonstrated that there is significant genetic heterogeneity within the same tumor. In fact, over 63 % of somatic mutations were not detectable across every region. Further – and more worryingly – the gene expression profiles that related to good and poor prognoses were often found within the same tumor [18], which may be due to a ‘big bang’ model of cancer growth [19]. A single biopsy may not be enough to reveal the correct prognosis regarding response and survival. In fact, tumor heterogeneity is constantly evolving, and in some cases, has been demonstrated to be an active process – for example, in glioblastoma cells, an EGFR-expressing population actively recruits healthy cells into the tumor and enhances the tumorigenicity of the entire cancer, suggesting evolutionary causes behind tumor growth [20].

Interestingly, there are parallels between rapidly proliferative, highly glycolytic cancer cells and stem cells, and a popular theory suggests that cancer may in fact be driven by a subset of cells that act like stem cells and serve as the nucleus for tumor growth [21, 22]. In healthy stem cell biology, differentiation status is linked to metabolic changes, where proliferating stem cells favor glycolysis as opposed to differentiation [23]. It is therefore possible that the cells that serve as the stem-like drivers of tumor growth could differentiate into the majority of the tumor mass, which has metabolic properties that are different from the original cancer stem cells. Cancer stem cells may represent only a tiny fraction of the actual tumor mass. In specific instances, the stem cells have been hypothesized to have the ability to drive proliferation within the tumor [24]. Similarly to stem cells, cancer stem cells can also become ‘quiescent’ [25], potentially allowing resistant cancer stem cells to survive and cause relapse. It is to be noted that the existence of cancer stem cells is controversial, but there is evidence in particular diseases that cancer stem cells may exist and may contribute to the understanding of a patient’s prognosis. For example, leukemic stem cells have been hypothesized to exist for many years [26].

An alternate theory also suggests that all cancer cells have a continuous spectrum of stem-like behavior, and this behavior may be influenced by microenvironmental cues [24, 27]. This would suggest that individual cells could, depending on the microenvironment, become more or less stem-like, which would naturally impact their metabolic profile. It has been demonstrated that cancer cells have the ability to regulate oxidative phosphorylation and glycolysis [28–30]. This regulation may be impacted by factors such as external glucose [28] or oxygen [31] availability. Since different portions of the tumors are exposed to different local microenvironments and therefore different local conditions (such as oxygen and glucose availability, exposure to cytokines or extracellular matrix proteins, and so on), the metabolic profile of the entire tumor may stand to be quite heterogeneous [24]. It has been shown in vitro that human breast cancer cells can revert to a healthy cell phenotype based on the impact of extracellular conditions alone [32, 33]. Along the same vein, a tumor may exhibit differential metabolic activity depending on the location of the cells in the tumor. It is known that the tumor core can become hypoxic as the tumor grows without access to vasculature [34]. As discussed later, there are changes in metabolic activity at the edge and core of tumors.

An additional factor that creates further nuance in our understanding of cancer cell metabolism is that glucose contributes not only to glycolysis, but to fatty acid production as well [35, 36]. Further, the cell may also produce ATP and thus energy from additional sources [37]. Specifically, when the total ATP turnover was studied for MCF-7 breast cancer cells, it was found that a number of different fuels contributed to total ATP production. The cells were fed ^14^C-labeled fuels in airtight chambers, and contribution to total ATP production was measured by measuring labeled ^14^CO_2_. It was found that these cells produced ATP through both oxidative phosphorylation and glycolysis, and that glucose, lactate, glutamine, palmitate and oleate all significantly contributed to the total ATP turnover [38].

**Fig. 1:**
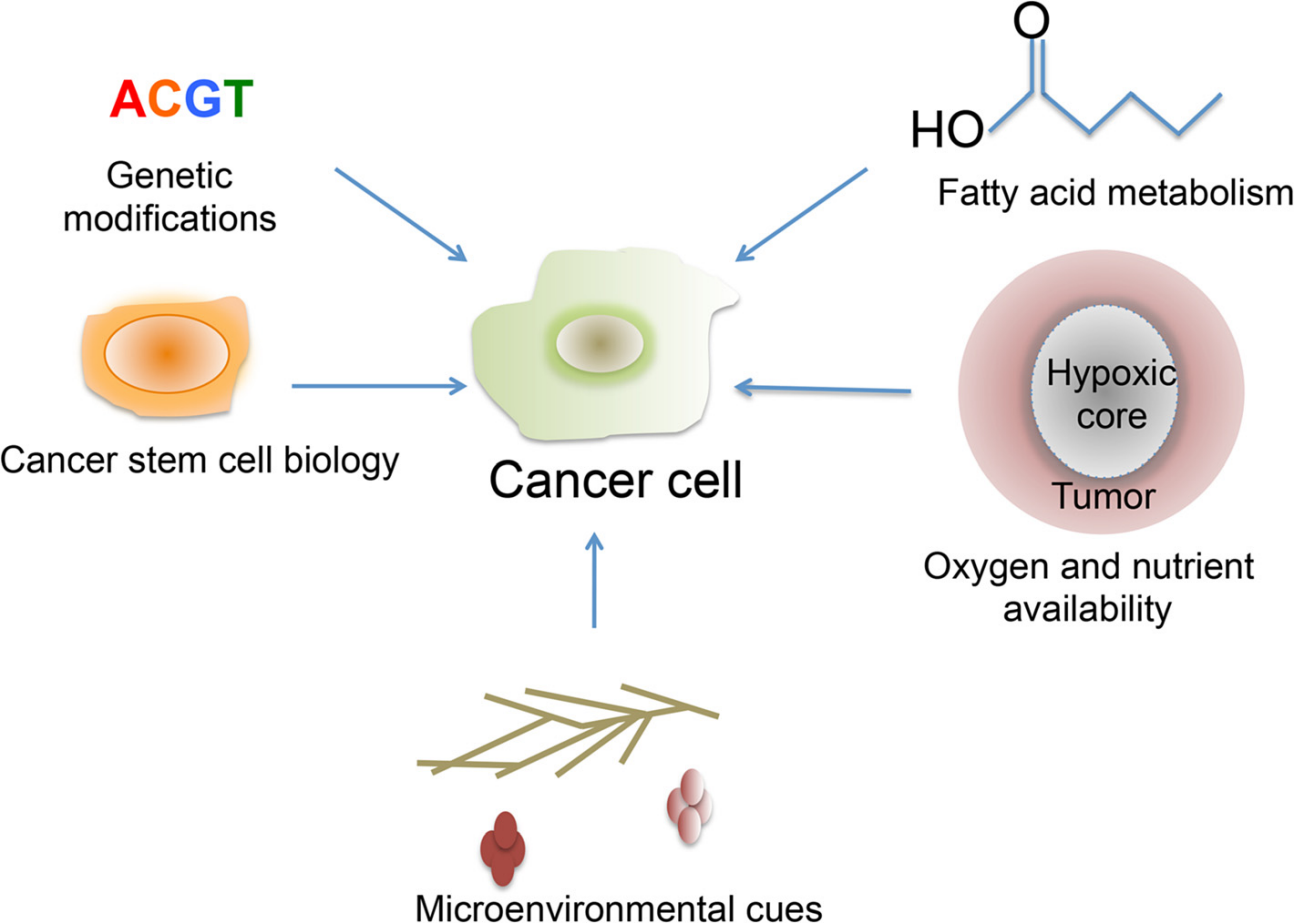
A schematic of factors that influence cancer metabolic heterogeneity

Because cancerous metabolic systems are so complex (Fig. 1), special tools are needed to measure and quantify metabolic activity both in vivo and in vitro. There are advantages and disadvantages to analyzing metabolic systems using both regimes. In vivo imaging techniques may be used in order to study metabolism in the native microenvironment. In vivo imaging can provide insight into both upstream and downstream effectors of metabolism since changes in DNA and proteins, energy storage, and glucose uptake itself can all be measured in this manner. Some of these techniques are, of course, limited by cost and feasibility. If any data is to be collected over time to determine treatment response, the invasiveness of the technique and the potential discomfort to the patient must also be taken into consideration.

### In vivo metabolic techniques

In the in vivo regime, the most common – and most clinically relevant – technique used for cancer metabolic imaging in specific is positron emission tomography (PET). PET is now a crucial aspect of many different types of cancer diagnoses, and is also increasingly used to monitor treatment response. PET is often combined with a diagnostic or low-dose companion CT scan, which uses X-rays to generate a three-dimensional image of the body. PET technology is most commonly used with the radiotracer FDG, which exploits the expanded glucose consumption of cancer cells to image tumors in a non-invasive manner. PET technology works by using a radioactive analog of glucose known as [^18^F]Fluorodeoxyglucose or FDG. Cancer patients are typically injected with 370 MBq (10 mCi) [39] of FDG. FDG is absorbed systemically by the cells of the body; however, it has been observed that a number of cancers will take up FDG more avidly than the surrounding normal tissue in the region of the cancer. FDG uptake is generally considered a viable surrogate of glucose utilization, and while the tracer does cause DNA damage, this damage is generally repaired within a few cell cycles [40, 41]. Cells take up FDG through glucose transporters, and GLUT1 and GLUT3 are often overexpressed in malignant cells [42, 43]. Unlike regular glucose, FDG is not completely metabolized within the cell. FDG is taken up and phosphorylated by hexokinase to FDG-6-phosphate, which accumulates in the cells, emitting a low level of radioactive emissions that can be detected and reconstructed to create a three-dimensional analysis of the tissue.

FDG-PET is an invaluable tool for cancer diagnosis, staging, and monitoring. The diseases it is typically used for include cancers of the breast, lung, head and neck, lymphoma, and sarcoma [44]. PET has been used since the 1980s [45, 46] to image glycolytic cells that preferentially take up FDG [47]. Glucose (and therefore FDG) uptake tracks closely with treatment response and tumor reduction. It has been found in a number of studies that PET scanning demonstrates decreases in FDG uptake over the course of a successful treatment. For example, breast cancer patients who underwent successful chemohormonotherapy demonstrated significant decreases in FDG uptake, even though the tumors did not decrease in size. The patients that did not respond to the drug did not show decreases in FDG uptake [48]. Similar results have also been observed in liver metastases [49] and squamous cell carcinoma [50]. Interestingly, there is some evidence that certain cancer cells may be able to distinguish between FDG and glucose, as evidenced by the fact that a healthy human being will excrete FDG but not glucose in urine [51].

While FDG-PET has undoubtedly changed the landscape of cancer management, the technique still has many limitations. FDG uptake can be influenced by a number of different factors. For example, some tumors, such as prostate cancers and certain lobular breast cancers [52], have inherently low FDG uptake. Glucose uptake may also be influenced by internal inflammatory processes and the presence of macrophages and granulation tissue [53]. It is possible for the tumor glucose uptake to be extremely heterogeneous, resulting in false positives or false negatives [54]. Small lesions and infiltrating disease beyond the gross tumor volume can be harder to detect due to PET’s spatial resolution, which is on the order of millimeters on average. Many tumors can be relatively small in size, and probing intratumoral heterogeneity is challenging given the fundamental limitations of PET. The detector width, positron range, and acollinearity of the annihilation photons all contribute to reduced spatial resolution [55]. Further, FDG-PET does not capture metabolic uptake from glutamine or other fuels.

**Fig. 2:**
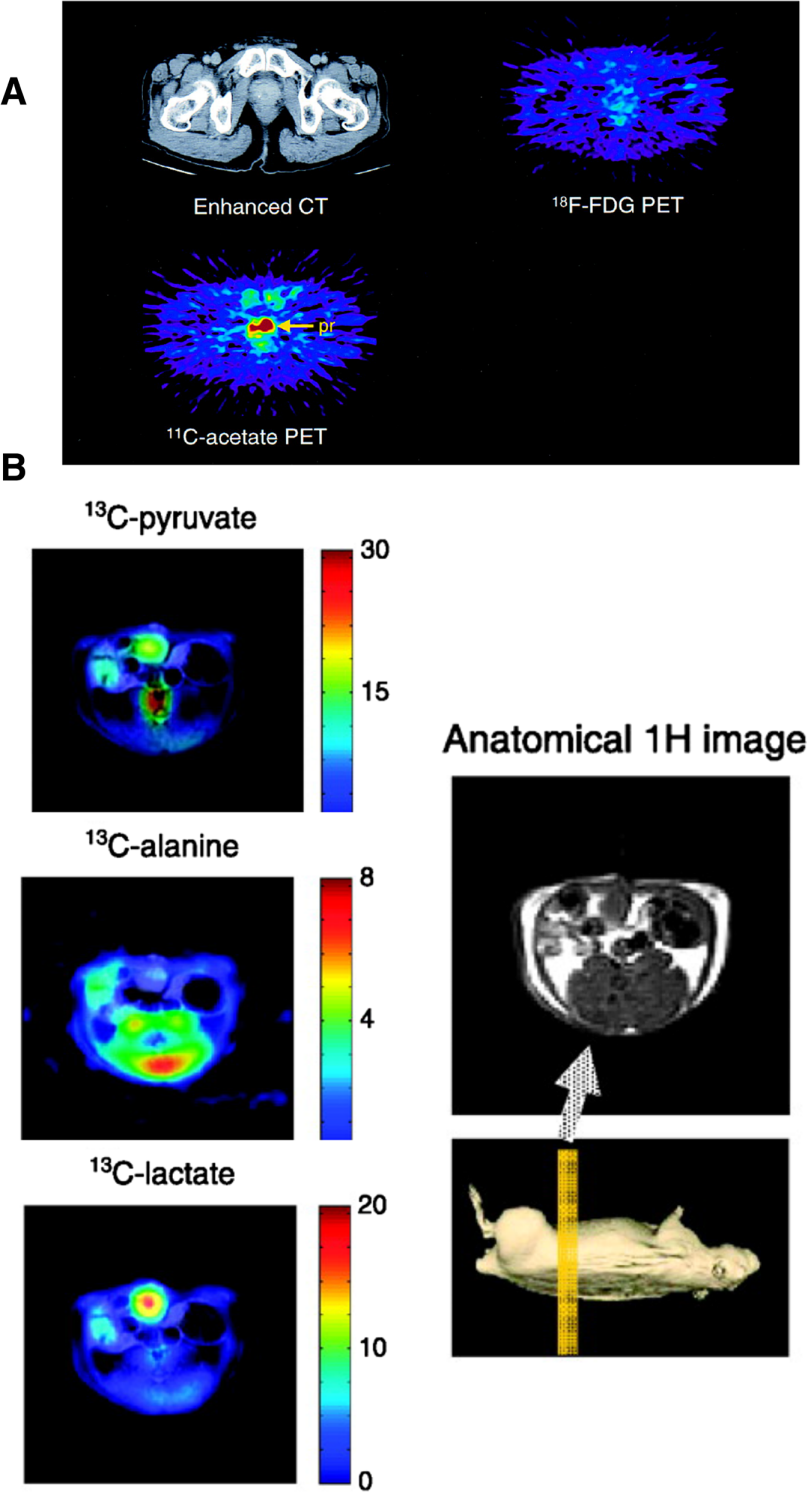
**a** PET/CT images comparing [^11^C] -acetate and FDG in a prostate cancer patient. This research was originally published in the Journal of Nuclear Medicine. Oyama N, Akino H, Kanamaru H, Suzuki Y, Muramoto S, Yonekura Y, Sadato N, Yamamoto K, Okada K: ^11^C-acetate PET imaging of prostate cancer. J Nucl Med 2002, 43:181-186. © by the Society of Nuclear Medicine and Molecular Imaging, Inc [58]. **b** Hyperpolarized MR imaging of hyperpolarized [^13^C] pyruvate and its conversion to [^13^C] alanine and [^13^C] lactate in vivo. Adapted by permission from the American Association for Cancer Research: Golman K, Lerche M, Pehrson R, Ardenkjaer-Larsen JH: Metabolic imaging by hyperpolarized 13C magnetic resonance imaging for in vivo tumor diagnosis. Cancer Research 2006, 66:10855-10860 [58]

In order to circumvent the limitations of FDG-PET, additional PET techniques are being developed with new compounds and isotopes that can target different steps in the metabolic pathway. A new radiotracer, ^18^F-*N*-(methyl-(2-fluoroethyl)-1H-[1–3]triazole-4-yl)glucosamine ([^18^F] NFTG), has been used to study another metabolic phenomenon in oncogenic cells – glycogenesis or glycogen storage. In times of energetic stress, cancer cells can accumulate glycogen in order to create a buffer against nutrient starvation [56]. To take advantage of this process, [^18^F] NFTG is used to image glycogen metabolism since it can be incorporated into stored glycogen. This compound has been demonstrated to accumulate during the nonproliferative state of cancer cells and demonstrates great promise in a preclinical study [56]. Additionally, glutamine-based metabolic activity can be studied using [^18^F] -(*2S,4R*)4-fluoroglutamine, a glutamine analog, which has also been shown to accumulate in preclinical tumor models [57]. This technique allows for the detection of non-glucose-based metabolic activity, which would be very useful in combination with or separately from FDG. [^11^C] choline has also been found to accumulate in certain cancers, because some cancers upregulate the production of choline kinase [58]. [^11^C] choline has been used to image prostate cancers [59], including the study of metastatic sites [60]. More research is needed to fully prove this compound’s diagnostic utility. An additional radiotracer is FSPG, which is a glutamate analogue [61]. Since fatty acids are also an important energy source for cancers, attention has been focused on the imaging of fatty acid oxidation pathways. For example, [^11^C] acetate-PET has been used to study a number of different types of cancer including prostate [58] and liver [62] cancers. [^11^C] acetate can be taken up by tumor cells and is used in fatty acid metabolism [63], and demonstrated marked uptake in prostate cancer as opposed to FDG [58], as shown in Fig. 2a. Additionally, a new compound, ^18^F-fluoro-pivalic acid or [^18^F] FPIA, an acetate analogue, has been successfully used in a preclinical study to image mouse models of various cancers, and shows promise for additional clinical studies [64].

PET scanning can also target other aspects of tumor growth, proliferation, and metabolism. Cell proliferation can be studied using 3'-deoxy-3'-[^18^F]fluorothymidine (FLT) [65] and [^11^C]thymidine [66]. Apoptosis has also been studied in a rat model using radiolabeled Annexin V [67]. Breast cancer has been studied using radioactive receptor-specific tracers [68, 69]. Tumor hypoxia may also be imaged using ^18^F-fluoromisonidazole (FMISO), and this type of PET can actually predict patient outcome after radiotherapy [70]. Amino acids such as [^11^C] methionine (MET-PET) [71, 72] and [^18^F] fluoroethyltyrosine (FET) have been used to image brain tumors [73]. While these novel tracers do not directly measure metabolism, they can be used in conjunction with FDG-PET to understand the impact of metabolic tumor heterogeneity on other aspects of tumor biology in real time.

Separately from PET, magnetic resonance spectroscopic imaging (MRSI) has recently been explored as a mechanism to monitor metabolism in vivo. MR spectroscopy has also been used to image metabolite distributions [74], including choline content, in cancer [75]. [^31^P] MRS has been used to monitor the levels of NAD+. This technique can be used in vivo and in vitro using tumor extracts, and has demonstrated that the administration of an NAD+ inhibitor does indeed map to decreases in NAD+ levels when monitored in a mouse mammary carcinoma model [76]. Interestingly, it has previously been demonstrated that hypoxic conditions correlate to glycolytic rate. In a murine mammary carcinoma model, ^13^C MR was used with ^1^H-^13^C cross-polarization to determine the rate of conversion of D-[1-^13^C]glucose into [3-^13^C]lactate. It was demonstrated that hypoxia dramatically lowers the glycolytic rate in this preclinical model [77]. A technique called “chemical exchange saturation transfer” or “CEST” MR imaging has been used in combination with natural D-glucose, and could demonstrate differences between two different types of breast tumors in a preclinical model [78]. This type of imaging, also termed ‘glucoCEST’, has been used to visualize and differentiate between human colorectal tumor xenograft phenotypes in a preclinical mouse model [79]. CEST MR can correlate with tissue redox state, and can predict NADH concentration and redox ratio in vivo in mouse breast cancer models [80].

More recently, hyperpolarized MRI using dynamic nuclear polarization (DNP) has been commonly used to probe in vivo metabolic heterogeneity in cancer. Hyperpolarized MRI is a technique whereby a particular compound to be injected is treated so that its nuclei spins are polarized to very high levels [81]. Although the half-life of the polarized state is typically quite short – on the order of dozens of seconds [82], advances in imaging permit the observation of these compounds as they are injected and metabolized. Many compounds that are part of the metabolic pathway have been evaluated in preclinical and clinical models, significantly enriching our understanding of in vivo metabolism. For example, using this approach, [^13^C] pyruvate was injected in a preclinical study with a rat model to track the conversion of pyruvate into lactate and alanine within tumors [83], as shown in Fig. 2b. It was found that the tumors demonstrated higher localized lactate content than healthy tissue. This type of detection has been suggested as a complement to PET scanning in a clinical setting [84] and may be an alternative method to study glucose utilization [85]. Hyperpolarized [1-^13^C]pyruvate has actually been used in a clinical study to image prostate cancer patients with promising initial results [86]. Hyperpolarized [U-^2^H, U-^13^C]glucose has also been used to study the conversion of glucose to lactate in mouse lymphoma and lung tumor models, also demonstrating that lactate was only found in the tumors and not in surrounding healthy tissue [87].

Hyperpolarized [^13^C] pyruvate can also be used to image the total hyperpolarized carbon in preclinical cancer models. For example, mice with low and high-grade prostate tumors were imaged using hyperpolarized MR, and were injected with hyperpolarized [^13^C] pyruvate. Lactate, pyruvate, and alanine were all imaged to determine the total hyperpolarized carbon in the mice. It was found that lactate and total hyperpolarized carbon levels all increased in broad correlation with tumor grade [88]. Similar techniques have also been used to image the conversion of fumarate into malate in a murine lymphoma model in vivo, demonstrating that the formation of hyperpolarized malate from fumarate may be a marker of tumor cell death [89]. Hyperpolarized choline can also be used to more sensitively image metabolites [90]. DNP-MRS has been used to study fructose uptake in a model of prostate cancer, where there was a dramatic difference in uptake and metabolism at the site of the tumor as compared to the surrounding tissue [91].

PET and MRI are thus both powerful indicators of in vivo metabolism. They are now beginning to be combined [92] in both clinical and preclinical settings [93]. In the context of tumor heterogeneity, this technique can allow for the simultaneous measurement of proliferation, necrosis, and inflammation [94]. Given the increasing library of PET radiotracers, and MRI methods, the combination of these two powerful techniques should serve to dramatically improve our understanding of tumor metabolic heterogeneity in the in vivo regime.

While these newer in vivo techniques may greatly enhance our understanding of the whole tumor metabolism, there are additional challenges in accurately imaging tumor metabolic heterogeneity in that some of these techniques require more translational validation before being used regularly in the clinic. Given the diversity of gene expression, the potential existence of cancer stem cells, and changes in microenvironment, the sensitivity of most of these techniques may not yet be high enough to provide a complete picture of tumor metabolism. In vitro techniques may therefore be powerful complements to in vivo systems in order to more finely study heterogeneous tumor cell metabolism.

### In vitro metabolic techniques

In vitro metabolic assays do not model the complexity of microenvironment or the entire biological context in the way that in vivo methods do. However, in vitro systems provide a level of manipulation and specificity that would be difficult to reproduce in vivo. In vitro experiments allow for a reductive approach, whereby it is easier to isolate the impact of specific factors on metabolism. For example, in an in vitro setting, it is possible to control levels of specific nutrients or oxygen provided to tumor cells in order to understand the impact of individual factors independently. Further, an in vitro setting allows for the study of tumor metabolic heterogeneity temporally within the same cell as well across multiple cells. In vitro systems therefore hold great promise as complements to in vivo metabolic monitoring techniques.

Some in vitro systems rely on the ‘grind and bind’ approach, where biopsies from patients or from preclinical models are obtained and then lysed to obtain proteins and metabolites from the tumor cells. This approach allows for the interrogation of the tumor in ways that would be impossible to reproduce in an in vivo setting. As previously mentioned, however, this method does carry with it the caveat that the biopsy specimen is now removed from its biological context and is a small part of a very heterogeneous whole. A single biopsy also only provides us with a snapshot of the tumor at a single point in time. Preservation and analysis must be done relatively rapidly such that there is no denaturing of proteins or destruction of key metabolites.

Where cells are preserved and cultured from within a tumor, it also becomes possible to conduct single cell analyses by dissociating tumors and isolating the cells of interest. Single-cell approaches are often low throughput, but allow for a more in-depth understanding of tumor heterogeneity. Again, studying cancer in a single-cell manner often necessarily involves the removal of the cell from its native context. However, if individual cells are cultured to be studied, a number of microenvironmental signals can be controlled, including temperature, oxygen content, extracellular matrix content, and so on. These techniques, when they are non-destructive, also allow us to track metabolism of the same cells over time.

In vitro methods have played a key role in our understanding of cancer cell metabolism. Some of Warburg’s observations were in fact based on levels of oxygen consumption in tumor cells and lactate secretions in ascites fluid [95]. In a biopsy sample, metabolites can be measured and quantified using imaging bioluminescence [96]. Interestingly, early experiments using this technique demonstrate much greater heterogeneity in tumor tissue than healthy tissue. Lactate is a byproduct of glycolysis and can accumulate in tumor tissue. Using bioluminescence imaging, the accumulation of lactate can be measured from biopsies, and has been correlated with increased metastases and lowered patient survival in ovarian cancer [97]. Similar results have also been found in biopsies of head-and-neck squamous cell carcinomas [98]. In the in vitro regime, mitochondrial activity has also been studied as a measure of hypermetabolic glycolysis in cancer cells [99]. Antibodies against mitochondrial activity have also been used as a measure of mitochondrial function in cancer [100]. In earlier studies, mitochondria from tumor cells were isolated and assayed for phosphate release [101], and it was found that mitochondria from cancer cells exhibited markedly lower ATPase activity. More recently, in vitro changes in mitochondrial activity were shown to be correlated with in vivo changes in glucose uptake as measured by FDG-PET [100]. Metabolites from lysed cells can also be correlated with gene expression profiles in cell lines [102], demonstrating, in this case, a key role for glycine metabolism in cancer cell line proliferation.

Newer techniques are also being developed to study the metabolome of cancer cells, providing detailed access on a scale that was not previously possible. This type of detailed analysis can be accomplished using the tools of mass spectrometry and stable isotope labeling. In order to effectively track metabolites through various cellular pathways, metabolic flux is characterized using compounds that have been labeled with stable isotopes such as ^13^C (natural abundance 1.1 %) or ^15^N (abundance 0.4 %). When cells are fed [^13^C] glucose, the downstream metabolites can be tracked using mass spectrometry or magnetic resonance techniques. Mass spectroscopy is generally destructive in that in order for the metabolites to be measured, the cell must be destroyed. However, this type of technology in combination with the comparatively less sensitive technique of NMR [103] is valuable in providing a complete picture of metabolism that also includes temporal information. This type of technique has been used clinically, where [^13^C] glucose was injected into human lung cancer patients, following which biopsies were taken in order to characterize metabolites from healthy as well as diseased tissue. It was found that many primary metabolites, such as lactate and citrate, were enhanced in the cancerous tissue [104]. In a preclinical study using SCID mice, a metabolite profile of healthy tissue was established, and then compared to a human lung carcinoma xenograft [105]. Stable isotope labeling has also been adapted to a microfluidics platform, which allows for multiplexed and quantitative measurement of cell metabolism and apoptotic processes in response to anticancer agents [106].

Metabolomics has also been used to assay metabolites in whole tissues and tumors. For example, capillary electrophoresis time-of-flight mass spectrometry was used to quantitatively measure metabolite levels in tumor and normal tissues obtained from colon and stomach cancer patients. It was found that levels of glucose were very low in both tumor tissues. Further, lactate and glycolytic intermediate concentrations were enhanced in these tissues [107]. Similarly, both high-resolution magic-angle spinning nuclear magnetic resonance (HR-MAS NMR) and gas chromatography mass spectrometry (GC/MS) were used to study metabolites from colorectal tumor biopsies, and to identify differences between metabolite levels in normal and cancerous tissues [108]. HR-MAS NMR has also been used on breast tissue from breast cancer patients to identify the upregulation of taurine- and choline-containing compounds in cancerous tissue [109]. Gas chromatography/time-of-flight mass spectrometry (GC-TOF MS) has also been used to study ovarian carcinomas and compare to borderline tumors of the ovary. A set of 291 metabolites were identified, and significant differences were found between the two groups [110].

Fluorescence techniques have been commonly used for in vitro metabolic measurements. For example, flow cytometry is an important in vitro method that has also been used in conjunction with in vivo FDG-PET data. DNA flow cytometry was able to track a correlation between the frequency of cells in S-phase and the amount of FDG taken up by the tumors. Interestingly, this data did not correlate with the histological grade of the tumors [111]. Another method called metabolic cytometry was used to follow metabolites within a single cell. In this method, capillary electrophoresis was used to draw individual cells up that were then lysed and individually analyzed for their metabolite profile using a fluorescent disaccharide substrate [112]. While this method is destructive and relatively low-throughput, this type of detailed analysis does provide a very complete metabolic picture of the cell. This type of analysis would be particularly valuable for rare cancerous cell populations such as cancer stem cells.

Fluorescence measurements can also be taken after tissue has been freeze-trapped in liquid nitrogen. Frozen tissue has been analyzed for metabolic redox state in 3D [113], and similar work has been done with the redox state of rat liver mitochondria [114]. More recently, this method has been used to quantify relative levels of chemical species in the metabolic pathway such as NADH and FAD in cervical tissue [115]. The redox ratios of cancerous tissue in breast cancer core biopsies have been found to be significantly different from healthy tissue in the same patient using this technique [116]. Similar work has been done in melanoma models [117] and a rat glioma model using NADH imaging as well as Pyro-2DG, an extrinsic GLUT-targeted photosensitizer [118]. The mitochondrial redox ratio can be a predictor of malignancy and can be correlated with other biomarkers in pancreatic cancer [119]. Advances in this technology mean that imaging can be performed in 3D and at extremely high spatial resolutions [120], and correlate tumor size and metastatic potential in breast cancer to heterogeneity of the mitochondrial redox state [121]. This technique can also be used to study metabolic alterations based on changes in oncogene expression [122]. The study of redox state can have significant implications on metastatic potential [123].

**Fig. 3:**
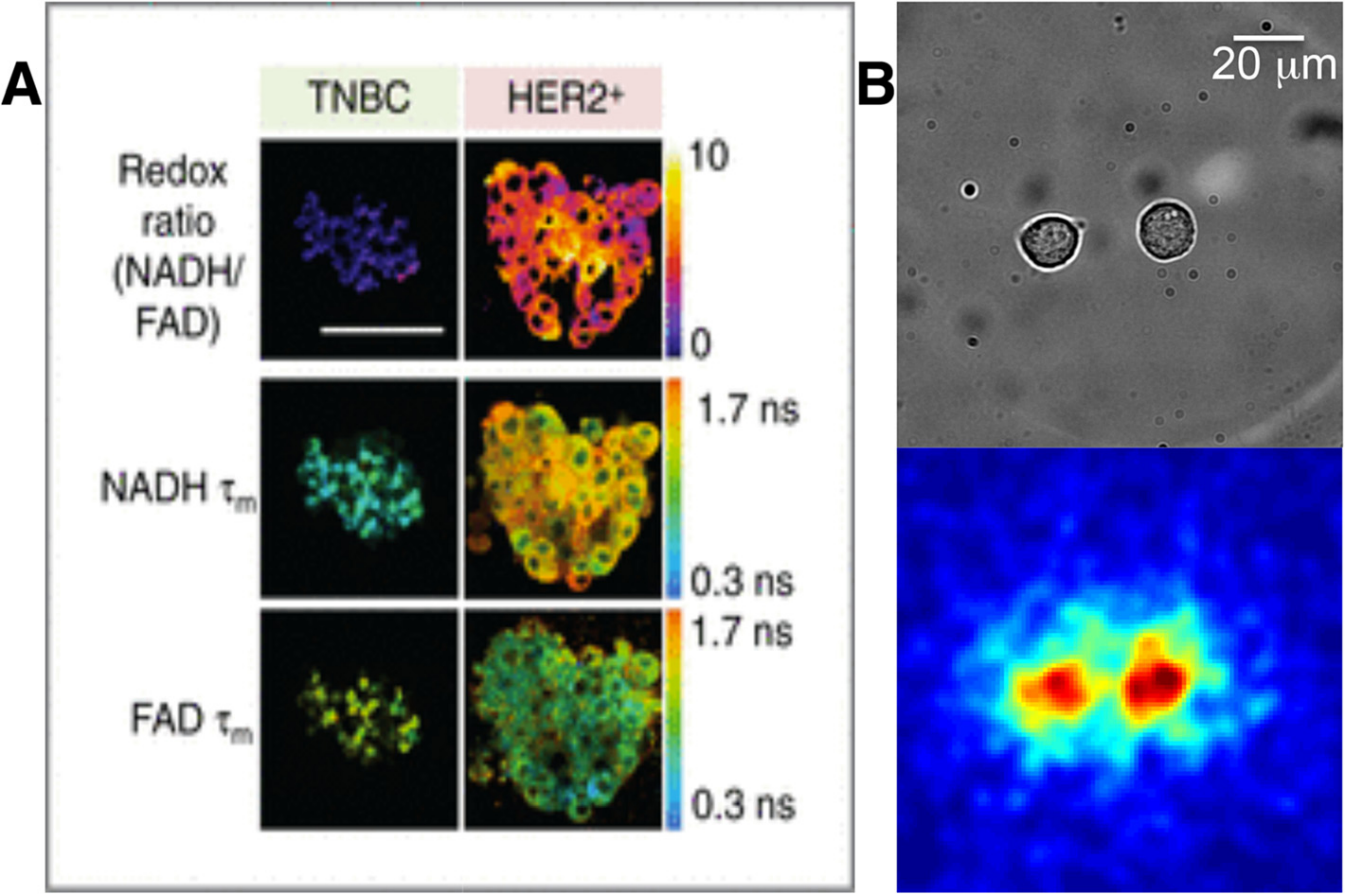
**a** Endogenous fluorescence images of NADH and FAD lifetimes in organoids derived from triple negative breast cancer (TNBC) and HER2+ tumors. Adapted by permission from the American Association for Cancer Research: Walsh AJ, Cook RS, Sanders ME, Aurisicchio L, Ciliberto G, Arteaga CL, Skala MC: Quantitative optical imaging of primary tumor organoid metabolism predicts drug response in breast cancer. Cancer Res 2014, 74:5184-5194 [124]. **b** Radioluminescence microscopy of single MDA-MB-231 human breast cancer cells that have taken up FDG

Interestingly, the endogenous fluorescent signals of metabolic coenzymes NADH and FAD have also been used to quantitate metabolism in vitro on a smaller scale. This technique has been used to study the efficacy of antitumor drugs in vitro, and then compare with xenograft-based in vivo drug responses [124]. This technique is sensitive enough for individual cell-based visualization and quantitation [125], as shown in Fig. 3a. Two-photon fluorescence measurements can also be used to study endogenous tissue fluorescence without any exogenous staining [126].

Single cell techniques can also be combined with microfluidic devices to characterize cell behavior. In one example, a microfluidic device could recreate the various microenvironmental conditions found in the liver, tumor, and marrow, and was able to effectively track individual cell drug metabolism [127].

Individual cell glucose consumption can also be elucidated using Raman, fluorescent, and radioactive glucose analogs in vitro. For example, a Raman-active analog of glucose called 3-O-propargyl-D-glucose (3-OPG) was recently synthesized, and an initial study suggests that 3-OPG can be measured when taken up in single cells using stimulated Raman scattering (SRS) [128]. 2-[N-(7-nitrobenz-2-oxa-1,3-diazol-4-yl) amino]-2-deoxy-D-glucose, also known as 2-NBDG, is a fluorescent glucose analog that has been used to study glucose uptake in breast cancer cells [129]. It is worth noting that the addition of a fluorescent moiety significantly changes the structure of the glucose molecule. Further, there is evidence to suggest that 2-NBDG follows pathways in use in quiescent rather than actively proliferative cancer cells, as with FDG [130].

Interestingly, FDG is also starting to be used for in vitro assays with single-cell level resolution. For example, FDG has been used in conjunction with a microfluidics device to study individual uptake in single melanoma cells [131]. FDG has also been used to characterize tumor glucose uptake heterogeneity within single cells. Because FDG is much more similar in size to actual glucose, it may be a better glucose analogue than 2-NBDG and other fluorescent analogues. When human breast cancer cells derived from a cell line were imaged after being incubated with FDG, considerable heterogeneity was found in these cells even when using a cell line [132, 133]. This type of technology allows us to visualize radiotracer uptake on a single cell level (Fig. 3b), which is an immensely valuable tool when monitoring heterogeneous tumor cell metabolism.

## Conclusion

In conclusion, tumor metabolism is a critical but as incompletely understood aspect of tumor biology. As previously discussed, cancer metabolism is considerably impacted by microenvironmental effects. There are genetic determinants of cancer metabolic biology, but it is possible that multiple genetic pathways contribute to an abnormal metabolic phenotype. Further, it is important not to oversimplify the metabolic activity of cancerous cells, as the cells follow multiple metabolic programs and utilize multiple fuels. A greater understanding of these processes will shape how we treat one of the greatest epidemics of our time.

Monitoring and studying cancer metabolism is therefore a critical step toward a greater understanding, better diagnostics, and effective therapies for cancer treatment. Both in vivo and in vitro regimes provide us with avenues to study tumor metabolism. In vitro techniques allow us to manipulate cells and study cells with striking levels of spatial and temporal resolution. In particular, single-cell techniques can provide us with detailed information about cancer cells in real time that were not possible in the past. In contrast, in vivo techniques allow us a detailed look at cells in their native microenvironment, although we are unable to follow metabolic pathways on a single-cell level. Together, however, these two regimes provide the ability to study tumor metabolic heterogeneity at different scales in order to create better treatments for the future.
